# 4-Amino-2,8-dimethyl-6*H*-pyrimido[1,2-*a*][1,3,5]triazin-6-one^1^

**DOI:** 10.1107/S1600536810027522

**Published:** 2010-07-17

**Authors:** Nikhil Sachdeva, Anton V. Dolzhenko, Geok Kheng Tan, Lip Lin Koh, Wai Keung Chui

**Affiliations:** a Department of Pharmacy, Faculty of Science, National University of Singapore, 18 Science Drive 4, Singapore 117543, Singapore; b Department of Chemistry, Faculty of Science, National University of Singapore, 3 Science Drive 3, Singapore 117543, Singapore

## Abstract

In the title compound, C_8_H_9_N_5_O, the mean planes through the pyrimidine and triazine rings form a dihedral angle of 2.83 (16)°. The amino group adopts a trigonal-planar configuration and forms an intra­molecular resonance-assisted N—H⋯O=C hydrogen bond with the carbonyl group. In the crystal, mol­ecules are linked *via* inter­molecular N—H⋯N hydrogen bonds into chains of *C*_2_^2^(6)[*R*_2_^2^(6)] motif. The molecules form two types of sheet parallel to (201) and (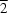
01), respectively.

## Related literature

For reviews on the synthesis and biological activity of fused 1,3,5-triazines see: Dolzhenko *et al.* (2006 ▶, 2008*a* ▶). For the synthesis and structural and biological investigations of pyrimido[1,2-*a*][1,3,5]triazines and their benzo-fused analogues, see Agasimundin *et al.* (1985 ▶); Dolzhenko *et al.* (2008*b* ▶, 2009**a* ▶,b* ▶). For the graph-set analysis of hydrogen bonding, see: Bernstein *et al.* (1995 ▶).
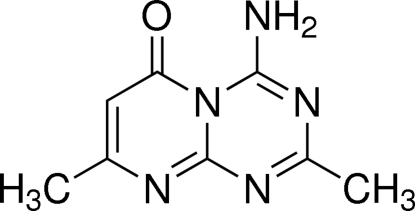


## Experimental

### 

#### Crystal data

C_8_H_9_N_5_O
*M* *_r_* = 191.20
Orthorhombic, 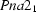

*a* = 11.1369 (19) Å
*b* = 18.913 (3) Å
*c* = 4.0311 (7) Å
*V* = 849.1 (3) Å^3^
*Z* = 4
Mo *K*α radiation
μ = 0.11 mm^−1^
*T* = 100 K
0.60 × 0.08 × 0.06 mm

#### Data collection

Bruker SMART APEX CCD diffractometer
Absorption correction: multi-scan (*SADABS*; Sheldrick, 2001 ▶) *T*_min_ = 0.938, *T*_max_ = 0.994
5774 measured reflections
1119 independent reflections
1019 reflections with *I* > 2σ(*I*)
*R*_int_ = 0.050

#### Refinement

*R*[*F* ^2^ > 2σ(*F* ^2^)] = 0.053
*wR*(*F* ^2^) = 0.117
*S* = 1.15
1119 reflections
137 parameters
1 restraint
H atoms treated by a mixture of independent and constrained refinement
Δρ_max_ = 0.33 e Å^−3^
Δρ_min_ = −0.25 e Å^−3^

### 

Data collection: *SMART* (Bruker, 2001 ▶); cell refinement: *SAINT* (Bruker, 2001 ▶); data reduction: *SAINT*; program(s) used to solve structure: *SHELXS97* (Sheldrick, 2008 ▶); program(s) used to refine structure: *SHELXL97* (Sheldrick, 2008 ▶); molecular graphics: *SHELXTL* (Sheldrick, 2008 ▶); software used to prepare material for publication: *SHELXTL*.

## Supplementary Material

Crystal structure: contains datablocks I, global. DOI: 10.1107/S1600536810027522/ez2220sup1.cif

Structure factors: contains datablocks I. DOI: 10.1107/S1600536810027522/ez2220Isup2.hkl

Additional supplementary materials:  crystallographic information; 3D view; checkCIF report

## Figures and Tables

**Table 1:** Hydrogen-bond geometry (Å, °)

*D*—H⋯*A*	*D*—H	H⋯*A*	*D*⋯*A*	*D*—H⋯*A*
N5—H51⋯O1	0.89 (4)	1.84 (4)	2.575 (3)	139 (3)
N5—H51⋯N1^i^	0.89 (4)	2.63 (4)	2.961 (4)	103 (3)
N5—H52⋯N4^i^	0.87 (4)	2.12 (4)	2.876 (4)	144 (3)

Symmetry code: (i) 
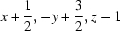
.

**Fig. 1.**
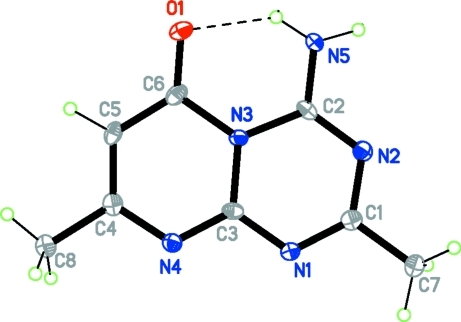
The molecular structure of the title compound showing the atomic numbering scheme. Displacement ellipsoids are drawn at the 50% probability level.

**Fig. 2.**
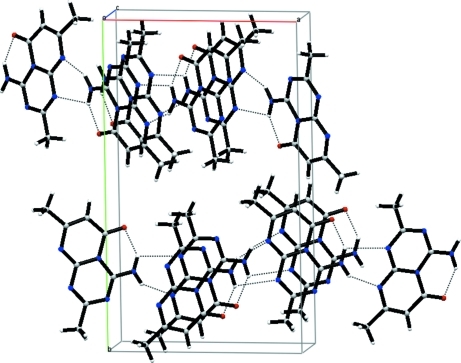
Crystal packing of the title compound, viewed down the c axis.

## supplementary crystallographic information

### Comment

Fused 1,3,5-triazines have been shown to possess a range of biological activities (Dolzhenko *et al.*, 2006; Dolzhenko *et al.*, 2008*a*). However, data on the synthesis and structure of pyrimido[1,2-*a*][1,3,5]triazines are limited (Agasimundin *et al.*, 1985; Dolzhenko *et al.*, 2009*b*). In continuation of our work on the synthesis, structural and biological investigation of pyrimido[1,2-*a*][1,3,5]triazines and their benzofused analogues (Dolzhenko *et al.*, 2008*b*; Dolzhenko *et al.*, 2009*a*,*b*), we report herein the molecular and crystal structures of 4-amino-2,8-dimethyl-pyrimido[1,2-*a*][1,3,5]triazin-6(5*H*)-one (Fig. 1 and 2).

The molecule of 4-amino-2,8-dimethyl-pyrimido[1,2-*a*][1,3,5]triazin-6(5*H*)-one is essentially planar with 0.0524 r.m.s. deviation for non-hydrogen atoms. The dihedral angle between the mean planes through the pyrimidine and triazine rings is 2.83 (16)°. The C—N bond distances at the bridgehead nitrogen atom *viz.* C2—N3 [1.4199 (37) Å], C3—N3 [1.4113 (36) Å] and C6—N3 [1.4488 (37) Å] are larger than typical values.The amino group adopts a trigonal planar configuration with atom N5 deviating by 0.0375 (229) Å from the C2/H51/H52 mean plane. The significantly shortened C2—N5 bond length [1.3114 (39) Å, the shortest C—N bond distance in the molecule] indicates a high degree of π-electron delocalization of the N5 atom with the heterocyclic system. The carbonyl group C6═O1 further extends this delocalization by resonance assisted N—H···O═C hydrogen bonding (Table 1).

In the crystal, the amino group acts as a hydrogen donor for intermolecular N—H···N hydrogen bonding with the nitrogen atoms N1 and N4 (Fig. 2), thereby forming extended chains with the C_2_^2^(6)[*R*_2_^2^(6)] hydrogen bond pattern (Bernstein *et al.*, 1995). The chains are organized in two types of sheets parallel to (201) and (201) planes, respectively.

### Experimental

The title compound was prepared by the cyclocondensation of 4-methyl-6-oxo-1,6-dihydropyrimidin-2-yl guanidine with triethyl orthoacetate. The details of the synthesis will be published elsewhere. Single crystals suitable for crystallographic analysis were grown by recrystallization from ethyl acetate.

### Refinement

All the H atoms attached to the carbon atoms were constrained in a riding motion approximation [0.95 Å for C_aryl_—H and 0.98 Å for methyl groups; *U*_iso_(H) =1.2*U*_eq_(C_aryl_) and *U*_iso_(H) = 1.5*U*_eq_(C_methyl_)] while the N-bound H atoms were located in a difference map and refined freely. In the absence of significant anomalous scattering effects 706 Friedel pairs have been merged.

### Crystal data

**Table:**

C_8_H_9_N_5_O	*D*_x_ = 1.496 Mg m^−^^3^
*M**_r_* = 191.20	Melting point: 545 K
Orthorhombic, *P**n**a*2_1_	Mo *K*α radiation, λ = 0.71073 Å
Hall symbol: P 2c -2n	Cell parameters from 614 reflections
*a* = 11.1369 (19) Å	θ = 2.8–26.8°
*b* = 18.913 (3) Å	µ = 0.11 mm^−^^1^
*c* = 4.0311 (7) Å	*T* = 100 K
*V* = 849.1 (3) Å^3^	Needle, colourless
*Z* = 4	0.60 × 0.08 × 0.06 mm
*F*(000) = 400	

### Data collection

**Table:**

Bruker SMART APEX CCD diffractometer	1119 independent reflections
Radiation source: fine-focus sealed tube	1019 reflections with *I* > 2σ(*I*)
graphite	*R*_int_ = 0.050
φ and ω scans	θ_max_ = 27.5°, θ_min_ = 2.2°
Absorption correction: multi-scan (*SADABS*; Sheldrick, 2001)	*h* = −13→14
*T*_min_ = 0.938, *T*_max_ = 0.994	*k* = −24→22
5774 measured reflections	*l* = −5→4

### Refinement

**Table:**

Refinement on *F*^2^	Primary atom site location: structure-invariant direct methods
Least-squares matrix: full	Secondary atom site location: difference Fourier map
*R*[*F*^2^ > 2σ(*F*^2^)] = 0.053	Hydrogen site location: inferred from neighbouring sites
*wR*(*F*^2^) = 0.117	H atoms treated by a mixture of independent and constrained refinement
*S* = 1.15	*w* = 1/[σ^2^(*F*_o_^2^) + (0.053*P*)^2^ + 0.4545*P*] where *P* = (*F*_o_^2^ + 2*F*_c_^2^)/3
1119 reflections	(Δ/σ)_max_ < 0.001
137 parameters	Δρ_max_ = 0.33 e Å^−^^3^
1 restraint	Δρ_min_ = −0.25 e Å^−^^3^

### Special details

**Table:**

Geometry. All esds (except the esd in the dihedral angle between two l.s. planes) are estimated using the full covariance matrix. The cell esds are taken into account individually in the estimation of esds in distances, angles and torsion angles; correlations between esds in cell parameters are only used when they are defined by crystal symmetry. An approximate (isotropic) treatment of cell esds is used for estimating esds involving l.s. planes.
Refinement. Refinement of *F*^2^ against ALL reflections. The weighted *R*-factor *wR* and goodness of fit *S* are based on *F*^2^, conventional *R*-factors *R* are based on *F*, with *F* set to zero for negative *F*^2^. The threshold expression of *F*^2^ > σ(*F*^2^) is used only for calculating *R*-factors(gt) *etc*. and is not relevant to the choice of reflections for refinement. *R*-factors based on *F*^2^ are statistically about twice as large as those based on *F*, and *R*- factors based on ALL data will be even larger.

### Fractional atomic coordinates and isotropic or equivalent isotropic displacement parameters (Å^2^)

**Table:**

	*x*	*y*	*z*	*U*_iso_*/*U*_eq_	
O1	0.61240 (19)	0.88225 (11)	−0.2411 (7)	0.0229 (6)	
N1	0.3706 (2)	0.71578 (13)	0.3214 (8)	0.0145 (6)	
N2	0.5341 (2)	0.67258 (13)	−0.0011 (7)	0.0137 (6)	
N3	0.4963 (2)	0.79702 (13)	0.0265 (7)	0.0119 (5)	
N4	0.3255 (2)	0.83294 (13)	0.3415 (7)	0.0138 (6)	
N5	0.6531 (2)	0.74865 (15)	−0.2904 (7)	0.0154 (6)	
H51	0.673 (3)	0.793 (2)	−0.326 (11)	0.018 (9)*	
H52	0.689 (3)	0.7098 (19)	−0.349 (11)	0.019 (9)*	
C1	0.4393 (3)	0.66472 (15)	0.2008 (8)	0.0134 (6)	
C2	0.5625 (2)	0.73778 (15)	−0.0888 (8)	0.0128 (6)	
C3	0.3965 (2)	0.78262 (15)	0.2314 (8)	0.0121 (6)	
C4	0.3465 (2)	0.90073 (15)	0.2475 (9)	0.0139 (6)	
C5	0.4418 (3)	0.91978 (16)	0.0552 (8)	0.0147 (6)	
H5	0.4531	0.9683	0.0019	0.018*	
C6	0.5246 (3)	0.86906 (15)	−0.0674 (8)	0.0155 (7)	
C7	0.4091 (3)	0.59100 (15)	0.3046 (10)	0.0191 (7)	
H7A	0.4559	0.5784	0.5017	0.029*	
H7B	0.4282	0.5582	0.1239	0.029*	
H7C	0.3232	0.5880	0.3562	0.029*	
C8	0.2543 (3)	0.95324 (15)	0.3617 (9)	0.0182 (7)	
H8A	0.1796	0.9460	0.2381	0.027*	
H8B	0.2840	1.0013	0.3224	0.027*	
H8C	0.2392	0.9467	0.5992	0.027*	

### Atomic displacement parameters (Å^2^)

**Table:**

	*U*^11^	*U*^22^	*U*^33^	*U*^12^	*U*^13^	*U*^23^
O1	0.0225 (11)	0.0152 (11)	0.0310 (14)	−0.0048 (8)	0.0116 (12)	0.0017 (11)
N1	0.0125 (11)	0.0128 (12)	0.0183 (14)	−0.0008 (9)	0.0027 (11)	0.0007 (11)
N2	0.0132 (11)	0.0131 (13)	0.0149 (13)	−0.0011 (9)	−0.0009 (11)	−0.0029 (11)
N3	0.0101 (10)	0.0113 (12)	0.0144 (13)	−0.0013 (8)	0.0024 (10)	−0.0011 (10)
N4	0.0116 (10)	0.0137 (12)	0.0162 (13)	−0.0004 (9)	0.0017 (11)	−0.0011 (11)
N5	0.0139 (12)	0.0123 (13)	0.0200 (15)	0.0017 (10)	0.0064 (12)	−0.0007 (12)
C1	0.0124 (12)	0.0152 (14)	0.0126 (15)	−0.0014 (11)	−0.0034 (12)	−0.0002 (13)
C2	0.0101 (12)	0.0167 (15)	0.0114 (14)	0.0023 (11)	−0.0002 (12)	−0.0034 (13)
C3	0.0108 (12)	0.0161 (14)	0.0096 (14)	−0.0022 (10)	0.0013 (12)	−0.0003 (13)
C4	0.0142 (13)	0.0136 (14)	0.0139 (15)	−0.0001 (10)	−0.0048 (12)	−0.0030 (14)
C5	0.0190 (14)	0.0085 (14)	0.0165 (16)	−0.0027 (11)	−0.0031 (13)	−0.0007 (12)
C6	0.0156 (13)	0.0122 (15)	0.0187 (17)	−0.0050 (11)	−0.0018 (13)	0.0009 (13)
C7	0.0215 (14)	0.0126 (14)	0.0232 (18)	−0.0004 (11)	0.0046 (15)	0.0005 (13)
C8	0.0182 (13)	0.0154 (15)	0.0209 (16)	0.0021 (11)	0.0018 (13)	−0.0010 (14)

### Geometric parameters (Å, °)

**Table:**

O1—C6	1.228 (4)	N5—H52	0.87 (4)
N1—C1	1.324 (4)	C1—C7	1.494 (4)
N1—C3	1.346 (4)	C4—C5	1.363 (4)
N2—C2	1.321 (4)	C4—C8	1.501 (4)
N2—C1	1.342 (4)	C5—C6	1.420 (4)
N3—C3	1.411 (4)	C5—H5	0.9500
N3—C2	1.420 (4)	C7—H7A	0.9800
N3—C6	1.449 (4)	C7—H7B	0.9800
N4—C3	1.314 (4)	C7—H7C	0.9800
N4—C4	1.357 (4)	C8—H8A	0.9800
N5—C2	1.311 (4)	C8—H8B	0.9800
N5—H51	0.89 (4)	C8—H8C	0.9800
			
C1—N1—C3	117.5 (3)	C5—C4—C8	122.2 (3)
C2—N2—C1	117.0 (2)	C4—C5—C6	121.7 (3)
C3—N3—C2	116.6 (2)	C4—C5—H5	119.2
C3—N3—C6	120.4 (2)	C6—C5—H5	119.2
C2—N3—C6	122.9 (2)	O1—C6—C5	125.3 (3)
C3—N4—C4	119.1 (3)	O1—C6—N3	120.9 (3)
C2—N5—H51	116 (2)	C5—C6—N3	113.8 (3)
C2—N5—H52	113 (2)	C1—C7—H7A	109.5
H51—N5—H52	130 (3)	C1—C7—H7B	109.5
N1—C1—N2	126.6 (3)	H7A—C7—H7B	109.5
N1—C1—C7	116.6 (3)	C1—C7—H7C	109.5
N2—C1—C7	116.8 (3)	H7A—C7—H7C	109.5
N5—C2—N2	119.7 (3)	H7B—C7—H7C	109.5
N5—C2—N3	118.6 (3)	C4—C8—H8A	109.5
N2—C2—N3	121.6 (3)	C4—C8—H8B	109.5
N4—C3—N1	117.4 (3)	H8A—C8—H8B	109.5
N4—C3—N3	122.1 (3)	C4—C8—H8C	109.5
N1—C3—N3	120.5 (3)	H8A—C8—H8C	109.5
N4—C4—C5	122.9 (3)	H8B—C8—H8C	109.5
N4—C4—C8	114.9 (3)		
			
C3—N1—C1—N2	0.9 (5)	C2—N3—C3—N4	−177.1 (3)
C3—N1—C1—C7	179.8 (3)	C6—N3—C3—N4	0.7 (4)
C2—N2—C1—N1	0.2 (5)	C2—N3—C3—N1	2.8 (4)
C2—N2—C1—C7	−178.8 (3)	C6—N3—C3—N1	−179.4 (3)
C1—N2—C2—N5	−178.8 (3)	C3—N4—C4—C5	−3.2 (5)
C1—N2—C2—N3	0.4 (4)	C3—N4—C4—C8	174.9 (3)
C3—N3—C2—N5	177.4 (3)	N4—C4—C5—C6	1.6 (5)
C6—N3—C2—N5	−0.3 (4)	C8—C4—C5—C6	−176.3 (3)
C3—N3—C2—N2	−1.8 (4)	C4—C5—C6—O1	−179.9 (3)
C6—N3—C2—N2	−179.5 (3)	C4—C5—C6—N3	1.0 (4)
C4—N4—C3—N1	−177.9 (3)	C3—N3—C6—O1	178.8 (3)
C4—N4—C3—N3	2.0 (4)	C2—N3—C6—O1	−3.6 (4)
C1—N1—C3—N4	177.5 (3)	C3—N3—C6—C5	−2.1 (4)
C1—N1—C3—N3	−2.4 (4)	C2—N3—C6—C5	175.5 (3)

### Hydrogen-bond geometry (Å, °)

**Table:**

*D*—H···*A*	*D*—H	H···*A*	*D*···*A*	*D*—H···*A*
N5—H51···O1	0.89 (4)	1.84 (4)	2.575 (3)	139 (3)
N5—H51···N1^i^	0.89 (4)	2.63 (4)	2.961 (4)	103 (3)
N5—H52···N4^i^	0.87 (4)	2.12 (4)	2.876 (4)	144 (3)

Symmetry codes: (i) *x*+1/2, −*y*+3/2, *z*−1.
